# Tumor localization by Prostate Imaging and Reporting and Data System (PI-RADS) version 2.1 predicts prognosis of prostate cancer after radical prostatectomy

**DOI:** 10.1038/s41598-023-36685-1

**Published:** 2023-06-21

**Authors:** Ayumi Fujimoto, Shinichi Sakamoto, Takuro Horikoshi, Xue Zhao, Yasutaka Yamada, Junryo Rii, Nobuyoshi Takeuchi, Yusuke Imamura, Tomokazu Sazuka, Keisuke Matsusaka, Jun-ichiro Ikeda, Tomohiko Ichikawa

**Affiliations:** 1 Department of Urology, Chiba University Graduate School of Medicine, 1-8-1 Inohana, Chuo-ku, Chiba, Chiba 260-8670 Japan; 2 Department of Radiology, Chiba University Hospital, Chiba, 260-8677 Japan; 3 Department of Pathology, Chiba University Hospital, Chiba, 260-8677 Japan; 4 Department of Diagnostic Pathology, Chiba University Graduate School of Medicine, Chiba, 260-8670 Japan

**Keywords:** Prostate cancer, Prostate

## Abstract

An improved reading agreement rate has been reported in version 2.1 (v2.1) of the Prostate Imaging and Reporting and Data System (PI-RADS) compared with earlier versions. To determine the predictive efficacy of bi-parametric MRI (bp-MRI) for biochemical recurrence (BCR), our study assessed PI-RADS v2.1 score and tumor location in Japanese prostate cancer patients who underwent radical prostatectomy. Retrospective analysis was performed on the clinical data of 299 patients who underwent radical prostatectomy at Chiba University Hospital between 2006 and 2018. The median prostate-specific antigen (PSA) level before surgery was 7.6 ng/mL. Preoperative PI-RADS v2.1 categories were 1–2, 3, 4, and 5 in 35, 56, 138, and 70 patients, respectively. Tumor location on preoperative MRI was 107 in the transition zone (TZ) and 192 in the peripheral zone (PZ). BCR-free survival was significantly shorter in the PZ group (p = 0.001). In the total prostatectomy specimens, preoperative PI-RADS category 5, radiological tumor location, pathological seminal vesicle invasion, and Grade Group ≥ 3 were independent prognostic factors of BCR. These four risk factors have significant potential to stratify patients and predict prognosis. Radiological tumor location and PI-RADS v2.1 category using bp-MRI may enable prediction of BCR following radical prostatectomy.

## Introduction

Prostate cancer was the second most common male cancer and the fifth leading cause of cancer death worldwide in 2020 (GLOBOCAN 2020)^1^. More than 1.4 million new cases and 375,000 deaths due to prostate cancer are estimated to occur globally per year. Radical prostatectomy remains one of the standard treatments procedure for localized prostate cancer, whereas active surveillance enhances clinical benefits for the low-risk group of prostate cancer^2^. However, pathological Grade Group (GG) may occasionally be overestimated or underestimated in patients who undergo radical prostatectomy for locally advanced prostate cancer at the initial biopsy. Misclassification of tumor risk at diagnosis leads to inadequate treatment, which is associated with inferior outcomes that include BCR and worse survival. The precise staging and estimation of malignancy are essential in the treatment strategies for localized prostate cancer.

In the diagnosis of prostate cancer, detection and localization of malignant lesions are performed using MRI^3^. The Prostate Imaging and Reporting and Data System (PI-RADS) was issued in 2012 by the European Society of Urogenital Radiology (ESUR) as a standardized guideline for the imaging and interpretation of prostate MRI. PI-RADS is also used in evaluating and reporting of prostate cancer on multiparametric MRI (mp-MRI)^4^. In 2015, the ESUR published PI-RADS v2.0^5^, followed by the revised PI-RADS v2.1 in 2019^6^. In PI-RADS v2.1, some cases changed the TZ category from 2 to 1 or 3. TZ assessment for category 2 lesions requires background assessments. TZ nodules that were 2 points in PIRADS v2 are downgraded to 1 point if the nodule is similar to the background. For a case with T2W score of 2, if the DWI score is 4 or 5, the overall PI-RADS category is upgraded from 2 to 3.

A previous study has indicated the equivalent utility of bi-parametric MRI (bp-MRI) and multi-parametric MRI (mp-MRI)^7^. The clinical value of PI-RADS v2.0 with bp-MRI and pathological Grade Group to predict BCR following radical prostatectomy also has been reported^8^. Patients with renal dysfunction or an allergy to contrast agent are not able to undergo dynamic contrast-enhanced (DCE) MRI. Investigation of a method for detection of prostate cancer in these patients is a pressing clinical issue. In this regard, bp-MRI can be used without contrast agent for imaging prostate tumors. Although PI-RADS v2.1 based on MRI has become the standard option for evaluation of the prostate, as yet there is limited evidence regarding PI-RADS v2.1 and the prediction of BCR after prostatectomy, particularly for bp-MRI. There is also evidence that tumor location influences the prognosis of localized prostate cancer^9^. Based on this evidence, we hypothesize that tumor location as well as MRI findings influence the outcome of radical prostatectomy.

Therefore, the aim of the present study was to examine the prognostic significance of the bp-MRI findings of prostate cancer for BCR, including location and PI-RADS v2.1 category.

## Results

### Patient characteristics

Table 1 lists the characteristics of the 299 patients that were analyzed in our study. Median follow-up was 49.8 months after radical prostatectomy, median PSA (ng/mL) was 7.6 ng/mL, and median age at operation was 67 years. Open radical prostatectomy (ORP), laparoscopic radical prostatectomy (LRP), and robotic-assisted radical prostatectomy (RARP) were performed in 33 (11.0%), 76 (25.4%), and 190 (63.5%) patients, respectively. Lymph node dissection was performed in 234 patients (78.3%). The PI-RADS v2.1 category of the index tumor was 1–2, 3, 4, and 5 in 35 (11.7%), 56 (18.7%), 138 (46.2%), and 70 (23.4%) patients, respectively. Of the 299 patients, 71 (23.7%) had extra-prostatic extension and 89 (29.8%) specimens had a positive resection margin. Seminal vesicle invasion was found in 28 (9.4%) of patients. Pathological Grade Groups 1, 2, 3, 4, and 5 were diagnosed in 23 (7.7%), 123 (41.1%), 93 (31.1%), 24 (8.0%), and 35 (11.7%) of patients, respectively (Table 1).

**Table 1:** Patient characteristics.

Characteristics	
Total patients	299
Median age at surgery (range), y	67 (46–77)
Median PSA (range), ng/mL	7.6 (2.3–87.16)
PI-RADS v2.0 score, n (%)	
1–2/3/4/5	66 (22.1%)/25 (8.4%)/138 (46.2%)/70 (23.4%)
PI-RADS v2.1 score, n (%)	
1–2/3/4/5	35 (11.7%)/56 (18.7%)/138 (46.2%)/70 (23.4%)
Radiological location (TZ/PZ)	107/192
Surgical approach n (%)	
Open/laparoscopic/robot-assisted	33 (11.0%)/76 (25.4%)/190 (63.5%)
Lymph node dissection, n (%)	234 (78.3%)
Pathological Grade Group, n (%)	
1/2/3/4/5	23 (7.7%)/123 (41.1%)/93 (31.1%)/24 (8.0%)/35 (11.7%)
Undiagnosed	1 (0.3%)
Extraprostatic extension (EPE1), n (%)	71 (23.7%)
Resection margin (RM+), n (%)	89 (29.8%)
Seminal vesicle invasion (SV+), n (%)	28 (9.4%)
Lymph node metastasis (N1), n (%)	4 (1.3%)
Median observation period (months)	49.8
Biochemical failure, n (%)	48 (16.1%)

*PSA* prostate-specific antigen, *PI-RADS* Prostate Reporting and Imaging and Data System, *TZ* transition zone, *PZ* peripheral zone.

Forty-eight patients (16.1%) experienced BCR during the observation period. Baseline PSA, PI-RADS category, radiological location, Pathological Grade Group, resection margin positive (RM+), and seminal vesicle invasion positive (SV+) results were significantly different between the two groups of patients with or without biochemical failure (Table 2).

**Table 2:** Clinical characteristics according to presence or absence of BCR.

Characteristic	With BCR	Without BCR	*p* value
No. patients (%)	48 (16.1%)	251 (83.9%)	–
Median baseline PSA (range), ng/mL	10.61 (4.15–47.35)	7.22 (2.3–87.16)	0.0026**
PI-RADS v2.1 category, n	1 (0), 2 (1), 3 (7), 4 (15), 5 (25)	1 (19), 2 (15), 3 (49), 4 (123), 5 (45)	< 0.0001**
PI-RADS v2.1 category 5, n (%)	25 (52.1%)	45 (17.9%)	< 0.0001**
Radiological location, TZ/PZ	8 (16.7%)/40 (83.3%)	99 (39.4%)/152 (60.6%)	0.0075**
Pathological Grade Group 3–5, n	40 (83.3%)	112 (44.6%)	< 0.0001**
Resection margin positive, n (%)	30 (62.5%)	59 (23.5%)	< 0.0001**
Seminal vesicle invasion, n (%)	15 (31.3%)	13 (5.18%)	< 0.0001**
Lymph node metastasis, n (%)	2 (4.2%)	2 (0.8%)	0.1763

*PSA* prostate-specific antigen, *PI-RADS* Prostate Reporting and Imaging and Data System, *BCR* biochemical recurrence, *TZ* transition zone, *PZ* peripheral zone. **p < 0.01.

### Cox proportional hazard models for BCR

Univariate Cox proportional hazard model identified the following as significant factors for BCR: initial PSA ≥ 7.6 ng/mL (p = 0.0319), extra-prostatic extension (EPE) positive (p < 0.0001), RM+ (p < 0.0001), SV+ (p < 0.0001), Pathological Grade Group ≥ 3 (p < 0.0001), lymph node metastases (p = 0.013), radiological tumor location at PZ (p = 0.002), and PI-RADS category 5 (p < 0.0001) (Table 3).

**Table 3:** Uni- and multivariate Cox proportional hazard models for BCR-free survival. Significant values are in bold.

Variable	Univariate			Multivariate		
	HR	95%CI	*p* value	HR	95%CI	*p* value
Age at surgery > 67 y	1.02	0.57–1.81	0.9489			
Initial PSA > 7.63	1.92	1.06–3.48	0.0319*	1.24	0.65–2.34	0.5169
EPE positive	5.39	2.88–10.08	< 0.0001**	1.31	0.57–2.99	0.7719
RM positive	4.59	2.49–8.46	< 0.0001**	2.17	1.01–4.68	0.083
SV invasion positive	8.54	4.54–16.06	< 0.0001**	2.65	1.26–5.58	0.0103*
Grade Group 3–5	6.40	2.86–14.31	< 0.0001**	2.82	1.20–6.64	0.0174*
Lymph node metastases	6.07	1.46–25.18	0.013*	2.31	0.49–10.9	0.2927
tumor location (PZ)	3.56	1.59–7.97	0.002**	2.96	1.23–7.11	0.0157*
PI-RADS category 5	4.20	2.36–7.44	< 0.0001**	2.8	1.48–5.29	0.0015**

*PSA* prostate-specific antigen, *EPE* extraprostatic extension, *RM* resection margin, *SV* seminal vesicle, *PZ* peripheral zone, *PI-RADS* Prostate Reporting and Imaging and Data System, *BCR* biochemical recurrence, *HR* hazard ratio, *CI* confidence interval. *p < 0.05; **p < 0.01.

Multivariate analysis identified the following as independent risk factors for BCR: Pathological Grade Group ≥ 3 (p = 0.0174), radiological tumor location at PZ (p = 0.0157), seminal vesicle invasion positive (p = 0.0103), and PI-RADS category 5 (p = 0.0015).

### Kaplan–Meier analysis

**Figure 1:**
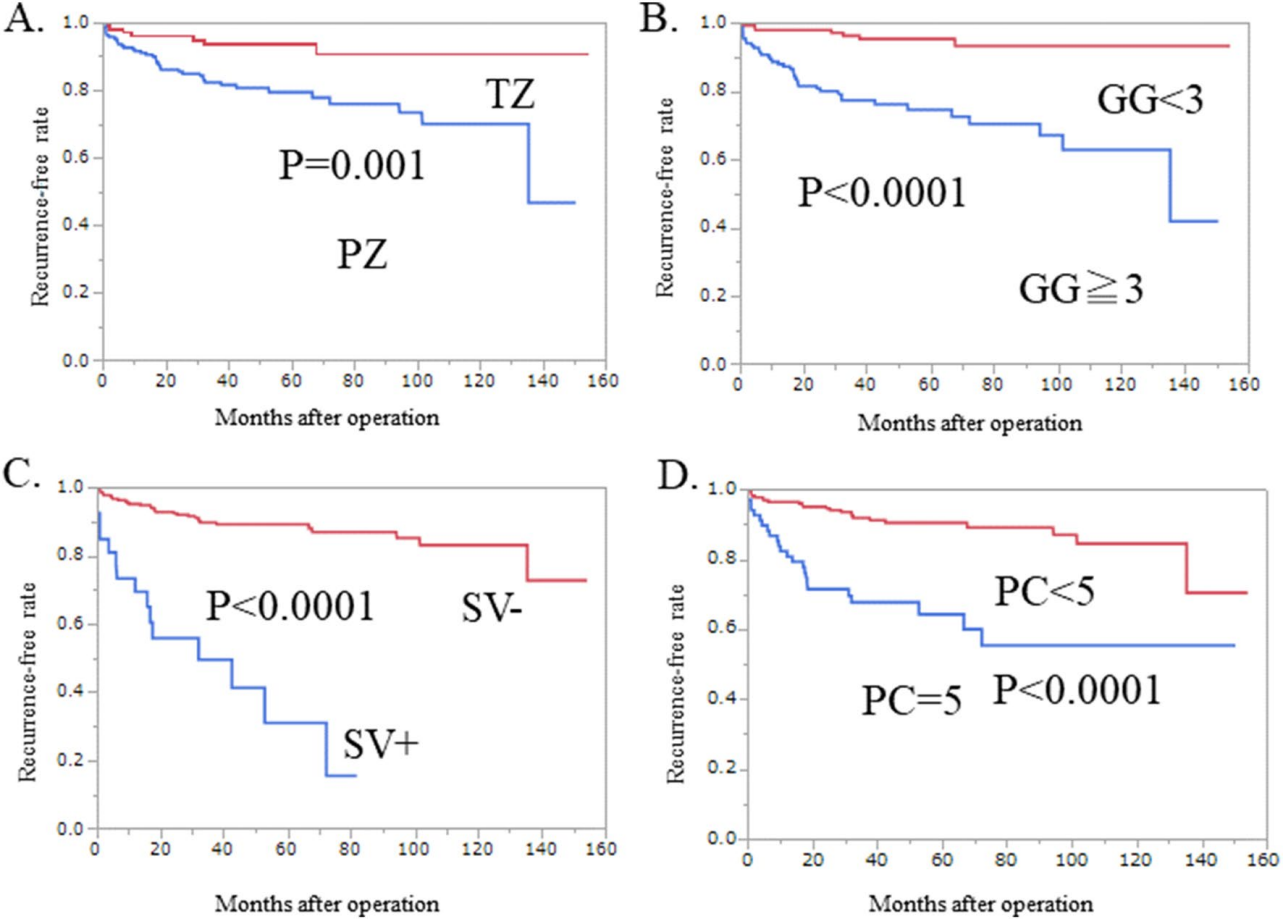
Kaplan–Meier analysis of factors identified as significant for BCR in the multivariate Cox proportional hazard model. (**A**) Radiological location. PFS in BCR was worse in tumors with radiological location in the PZ than in the TZ (p = 0.001). (**B**) Pathological Grade Group (GG). (**C**) Seminal vesicle invasion. (**D**) PI-RADS category (PC) 5. Tumors with Grade Group (3–5), seminal vesicle invasion (SV+), and PI-RADS category 5 had worse PFS in BCR (p < 0.0001, p < 0.0001, and p < 0.0001, respectively) compared with Grade Group (1–2), SV-, and PI-RADS category (1–4).

We performed Kaplan–Meier analysis to analyze factors identified as significant in the multivariate Cox proportional hazard model, which were radiological location at PZ (p = 0.001) (Fig. 1A), pathological Grade Group 3 (Fig. 1B), seminal vesicle invasion (SV+) (Fig. 1C), and PI-RADS category 5 (Fig. 1D) (all p < 0.0001). We built our original prediction model based on these four risk factors accordingly.

### Prognostic model for BCR using v2.1

**Figure 2:**
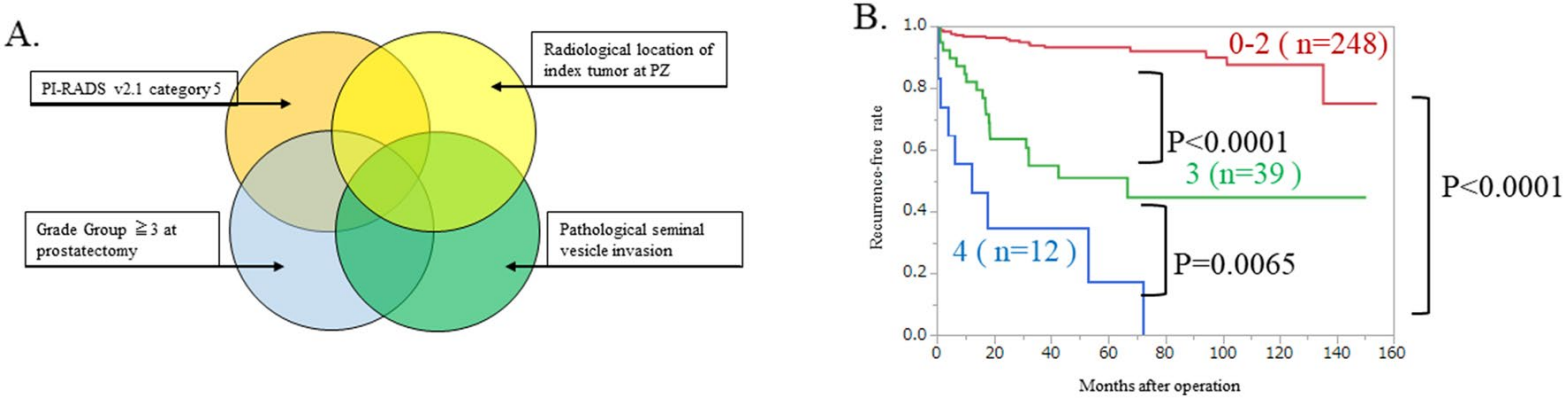
Novel prognostic model for BCR that combines PI-RADS v2.1 and clinical factors. (**A**) Novel prognostic model for BCR. The scoring system classifies the risk category according to the four factors predictive of BCR after radical prostatectomy. (**B**) Kaplan–Meier curve according to the novel prognostic model. The total score is the summed score of all positive factors (one point each). We divided the patients into three groups according to the summed score. Patients with a score of 0–2 were defined as the low-risk group (n = 248), those with 3 points as the intermediate-risk group (n = 39), and those with 4 points as the high-risk group (n = 12). Risk classification significantly differentiated the PFS of BCR between the high- and intermediate-risk, between the intermediate- and low-risk, and between the low- and high-risk groups (p = 0.0065, p < 0.0001, p < 0.0001).

We propose a new scoring system that classifies the risk categories by the four factors (Pathological Grade Group ≥ 3, radiological location at PZ, seminal vesicle invasion, and PI-RADS category 5) predictive of BCR after radical prostatectomy (Fig. 2A). One point is assigned for each positive factor, and the points are summed to give the total score. We divided the patients into three groups according to the summed score, as follows: score 0–2, low-risk group; 3 points, intermediate-risk group; and 4 points, high-risk group. There were 248 (82.9%), 39 (13.0%), and 12 (4.0%) patients in the low-, intermediate-, and high-risk groups, respectively. The Kaplan–Meier method was used to evaluate prognosis. Prognosis for BCR was the worst in the high-risk group. This novel prognostic model for BCR, which takes into account PI-RADS v2.1 as well as clinical factors, enables differentiation of patients according to risk factors for PFS between high- and intermediate-risk (p = 0.0065), intermediate- and low-risk (p < 0.0001), and low- and high-risk groups (p < 0.0001) (Fig. 2B).

### Radiological location as a preoperative predictive factor

Radiological location in the PZ was a worse prognostic factor than in the TZ (Fig. 1A). Patients with tumors in the radiological TZ had a lower BCR rate (7.5%) compared with those in the radiological PZ (20.8%) (p = 0.0075) (Table 2). We divided patients into two groups according to the radiological location (radiological TZ and PZ groups).

The univariate Cox proportional hazard model found no factors of significance for BCR in the TZ group, whereas the PZ group showed significant differences in terms of EPE positive (p < 0.0001), RM positive (p < 0.0001), SV positive (p < 0.0001), GG ≥ 3 (p = 0.0003), lymph node metastases (p = 0.0388), and PI-RADS category 5 (p < 0.0001). Furthermore, multivariate analysis identified RM positive (p = 0.0219), SV positive (p = 0.0114), Grade Group ≥ 3 (p = 0.0201), and PI-RADS category 5 (p = 0.0001) as independent risk factors (Table 4).

**Table 4:** Difference in the predictive factors between the radiological location. Significant values are in bold.

Variable	PZ						TZ		
	Univariate			Multivariate			Univariate		
	HR	95% CI	*p* value	HR	95% CI	*P* value	HR	95% CI	*p* value
Age at surgery > 67 y	1.01	0.54–1.89	0.9653				1.03	0.23–4.65	0.9628
Initial PSA > 7.63	1.64	0.86–3.11	0.1307				3.00	0.58–15.5	0.1895
EPE positive	5.49	2.74–11.03	< 0.0001**	1.38	0.57–3.32	0.478	2.46	0.45–13.4	0.4922
RM positive	4.97	2.46–10.04	< 0.0001**	2.63	1.15–6.03	0.0219*	1.52	0.29–7.83	0.8832
SV invasion positive	7.78	3.96–15.3	< 0.0001**	2.72	1.25–5.90	0.0114*	4.83	0.57–41.2	0.1501
Grade Group 3–5	6.75	2.40–19.0	0.0003**	3.61	1.22–10.6	0.0201*	2.32	0.47–11.5	0.3032
N+	4.52	1.08–18.9	0.0388*	2.57	0.54–12.3	0.2381	–	–	–
PI-RADS category 5	5.63	3.00–10.6	< 0.0001**	3.85	1.93–7.70	0.0001**	0.63	0.76–5.27	0.6729

*PSA* prostate-specific antigen, *EPE* extraprostatic extension, *RM* resection margin, *SV* seminal vesicle, *N+*: ymph-node positive, *PI-RADS* Prostate Reporting and Imaging and Data System, *PZ* peripheral zone, *TZ* transition zone. *p < 0.05; **p < 0.01.

It appears that preoperative PI-RADS location can predict the incidence of postoperative BCR. Patients with tumor in the radiological PZ region are more likely to suffer BCR if this finding is combined with the above four factors (RM positive, SV positive, Grade group ≥ 3, and PI-RADS category 5) following radical prostatectomy.

### Effect of radiological localization on efficacy of predictive factors

**Figure 3:**
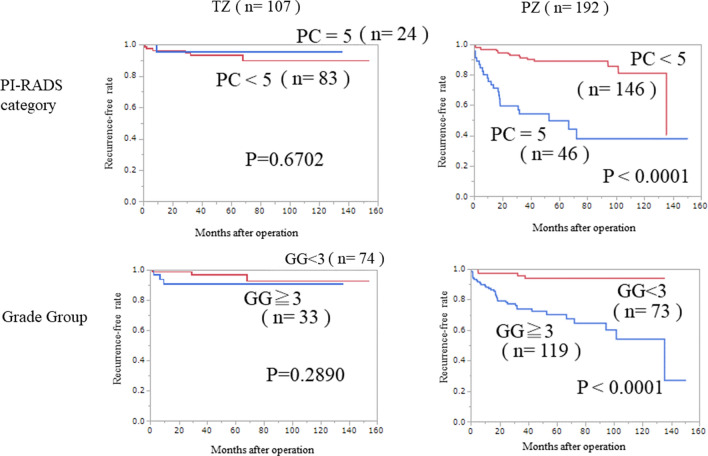
Kaplan–Meier analysis of efficacy of predictive factors according to radiological location. In PZ tumors, PI-RADS category 5 and Grade group ≥ 3 were significant predictive factors of BCR (p < 0.0001 and p < 0.0001, respectively). In TZ tumors, PI-RADS category 5 and Grade group ≥ 3 were not predictive of BCR (p = 0.6702 and p = 0.2890, respectively). These findings illustrate that the efficacy of GG ≥ 3 and PI-RADS category 5 differ according to the radiological location of the tumor.

Tumors located in the TZ had a better prognosis for BCR (Table 4). Kaplan–Meier analysis among the radiological PZ tumors identified PI-RADS category 5 (p < 0.0001) and Grade Group ≥ 3 (p < 0.0001) as significant factors predictive of BCR. For tumors located in the TZ, neither of these factors was predictive of BCR (p = 0.6702 and p = 0.2890, respectively) (Fig. 3).

These results indicate that Grade group ≥ 3 and PI-RADS category 5 could be used to assess the likely occurrence of BCR in PZ tumors, and show that the efficacy of the predictive factors varies according to the radiological location.

## Discussion

The present study is the first to report that BCR after radical prostatectomy can be predicted by preoperative MRI tumor location evaluated by PI-RADS v2.1. Our results showed that zonal location of the tumor on preoperative MRI was a significant predictor of BCR. Based on the factors remaining by multivariate analysis for prediction of BCR, we propose a novel risk-classification model based on the following: PZ lesion on MRI, Pathological Grade Group ≥ 3, seminal vesicle invasion, and PI-RADS category 5. Classification of patients into the low-risk (0–2 points), intermediate-risk (3 points), and high-risk (4 points) groups predicted the prognosis of localized prostate cancer patients with statistically significant accuracy. The proposed risk classification system may contribute to the development of treatment strategies for localized prostate cancer.

Takahashi et al. reported that in radical prostatectomy specimens of Japanese patients, approximately 40% of prostate cancer originated in the TZ^10^. Compared to Caucasian men, Japanese patients had a greater incidence of TZ cancer. The pathological characteristics of TZ and PZ cancer are similar except for pathological T stage in the case of autopsy and cystoprostatectomy for bladder cancer^11^. TZ cancers are associated with decreased odds of adverse pathological findings and demonstrate improved recurrence-free survival. These favorable outcomes appear to be the result of different tumor biology^12^. Understanding the biology of tumors originating in different prostate zones will enable zone-specific therapies^13^. The present study revealed that for prediction of BCR, the efficacy of Grade group ≥ 3 and PI-RADS category 5 differed between the radiological TZ and PZ. This risk criterion may predict BCR after radical prostatectomy and enable optimization of zone-specific therapeutic strategy. As discussed in a previous report^13^, zone-specific strategies may be considered when choosing between active surveillance, radical prostatectomy, and extended lymph node dissection in patients with Gleason Score and T stage in the same category but in different location. The rationale for the zone-specific strategy may be explained by the difference in the genetic background and biomarker between TZ and PZ, which will lead to the difference in the therapeutic response and prognosis^13^.

Previous studies have shown that seminal vesicle invasion and extraprostatic extension predict BCR after radical prostatectomy are related to predictive factors^14–16^. A positive surgical margin affects the incidence of BCR^17,18^. BCR risk is significantly higher for posterior-positive surgical margin than for other positive surgical margins^19^. Broad and anterior positive surgical margin has the highest risk of recurrence after radical perineal prostatectomy^20^. Prognosis was worse in the case of positive seminal vesicle invasion on preoperative MRI compared with negative seminal vesicle invasion^21^.

Several reports have evaluated oncological outcomes in patients with negative mp-MRI. Vinayak reported that patients with negative MRI findings (PI-RADS v2.0 score ≤ 2) who underwent radical prostatectomy had oncological outcomes comparable with positive MRI findings (PI-RADS v2.0 score ≥ 3) in terms of clinically significant prostate cancer rates, positive surgical margins, and BCR rates^22^. Shin et al. assessed patients with PI-RADS categories 4–5 on preoperative MRI who underwent prostatectomy and concluded that prognosis was predicted by the location of the lesion on preoperative MRI^23^.

In the present study, we analyzed patients with PI-RADS categories 1–5, not just categories 4–5. We found that prognosis was predicted by tumor location in PI-RADS v2.1 category 5 by MRI. To the best of our knowledge, this is the first study to report the ability of zonal location on preoperative MRI to predict post-operative BCR of prostate cancer using PI-RADS v2.1.

### Differences in evaluation between PI-RADS v2.0 and PI-RADS v2.1

There are three significant differences between PI-RADS v2.1 and v2.0 in evaluating scoring. First, the definitions of scores 1 and 2 have been revised for TZ lesions on T2WI. Second, on evaluating the total score in TZ, a DWI score of 4 or 5 elevates the overall PI-RADS assessment category from 2 to 3 for lesions receiving a T2WI score of 2. Third, the definitions for DWI scores of 2 and 3 have been revised for lesions located in TZ/PZ. As PI-RADS v2.1 improves inter-reader reproducibility, these revisions may contribute to increased diagnostic performance^6,24^. We have previously reported that bp-MRI and Grade Group predict BCR after radical prostatectomy^8^. In the present study, we analyzed the predictive ability of location on preoperative MRI and evaluation using the new categorization in PI-RADS v2.1 in a large number of patients who underwent radical prostatectomy. In our study, changing to the PI-RADS v2.1 criteria resulted in a change in classification for 40 of the 299 patients. The data of these 40 patients are summarized in Supplementary Table 1.

### Limitations

There are several limitations of this study. First, the number of patients analyzed was relatively limited and the evaluations were performed retrospectively. We plan to confirm our results in multi-institutional and prospective settings. Second, the median follow-up period was 49.8 months, and thus assessment related to survival was inadequate. It is necessary to assess oncological outcomes in a longer term. Third, surgery was performed mainly by three surgeons. The differences in prognosis may have been affected by the surgeons’ skills. Finally, patients of a single Asian race were investigated in our study. The incidence of and deaths due to prostate cancer are lower in the Asian population than in the Western population^25^, which might have some impact on the generalizability of our results.

## Conclusion

To the best of our knowledge, this is the first report to evaluate the risk of BCR by radiological tumor location by PI-RADS v2.1 category on preoperative MRI and by pathological diagnosis. We propose a novel risk-classification model based on the following independent risk factors: PZ location on MRI, Pathological Grade Group ≥ 3, seminal vesicle invasion, and PI-RADS category 5. This risk model could be applied to constructing and optimizing treatment strategies for patients with localized prostate cancer.

## Materials and methods

Clinical data from 299 patients who had undergone radical prostatectomy at Chiba University Hospital between 2006 to 2018 were retrospectively investigated. Ethics declaration: The study was approved by the Research ethics committee of the graduate school of medicine, Chiba University (approval number 2718). Informed consent was obtained from all participants and/or their legal guardians. The present study was conducted in accordance with ethical standards that promote and ensure respect and integrity for all human subjects and the Declaration of Helsinki. All experiments were performed in accordance with relevant named guidelines and regulations. The clinical factors of Gleason score, pathological features, and clinical tumor location were obtained from the patients’ medical records. Radical prostatectomy was performed by one of three surgical approaches (open, laparoscopic, and robot-assisted). Lymph node dissection was performed in 234/299 patients (78.3%). All patients underwent preoperative MRI followed by prostate biopsy and total prostatectomy.

We compared each patient’s scores for Prostate Imaging Reporting and Data System (PI-RADS) version 2.1 and version 2.0, based on bp-MRI. Overall survival and BCR-free survival were evaluated by the Kaplan–Meier method.

### Definition of PSA progression

Using the definition of the Prostate Cancer Clinical Trial Working Group 2 (PCWG2)^26^, we defined BCR as an elevation in PSA of ≥ 0.2 ng/mL after radical prostatectomy, which was confirmed in two consecutive measurements obtained at least 2 weeks apart. We defined the operation date as the date of PSA failure if PSA was ≥ 0.2 ng/mL after radical prostatectomy.

### MRI protocol

All enrolled patients underwent prostate MRI at 3 T prior to prostate biopsy. MRI was obtained with T1-weighted, T2-weighted, and diffusion-weighted imaging (DWI), and apparent diffusion coefficient maps were generated with b values of 0 and 1000 s/mm^2^. We used a high b-value (b = 2000) for DWI. bp-MRI comprised T2-weighted imaging and DWI. The radiologist used both bp-MRI and the apparent diffusion coefficient maps to determine the PI-RADS score.

### PI-RADS v2.1

The PI-RADS scores were evaluated on non-contrast-enhanced bp-MRI by one radiologist (T.H.) with over 10 years of experience in diagnostic radiology. Using the scoring method of PI-RADS v2.1, each patient’s score was recorded using a 5-point scale (1–5) and the zonal location. PI-RADS v2.1 was designed to improve detection, location, characterization, and risk stratification in patients with suspected cancer in treatment-naive prostate glands, with the overall objective of improving outcomes for patients. The changes incorporated in PI-RADS v2.1 were revised scoring of DWI in all zones in categories 2–3, and scoring of the overall assessment category in TZ. In TZ, a DWI score of 4 or 5 elevates the overall PI-RADS assessment category from 2 to 3 for lesions that receive a T2W score of 2. PI-RADS v2.1 states that T2-weighted images should be evaluated in the axial plane and in at least one additional orthogonal plane^27^.

### Statistical analysis

We performed univariate and multivariate Cox proportional hazard analyses to evaluate hazard ratios for BCR-free survival. Cut-offs of continuous variables were selected according to median values. Hazard ratios and 95% confidence intervals were derived. Kaplan–Meier methods were used for survival analysis. Statistical analysis was performed using JMP 14.2.0 (SAS Institute, Cary, NC, USA). Significance was considered at *p* < 0.05.

## Supplementary Information

Supplementary Table 1.

## Data Availability

The data sets used and analyzed in the current study are available from the corresponding authors upon reasonable request.
