# Supplementary material for: Tumor localization by Prostate Imaging and Reporting and Data System (PI-RADS) version 2.1 predicts prognosis of prostate cancer after radical prostatectomy
Source: Sci Rep. 2023 Jun 21;13:10079. doi: 10.1038/s41598-023-36685-1 (PMC10284848; doi:10.1038/s41598-023-36685-1)
Supplement: Supplementary file 1 — Supplementary Table 1. [file 41598_2023_36685_MOESM1_ESM.pptx]

## Slide 1
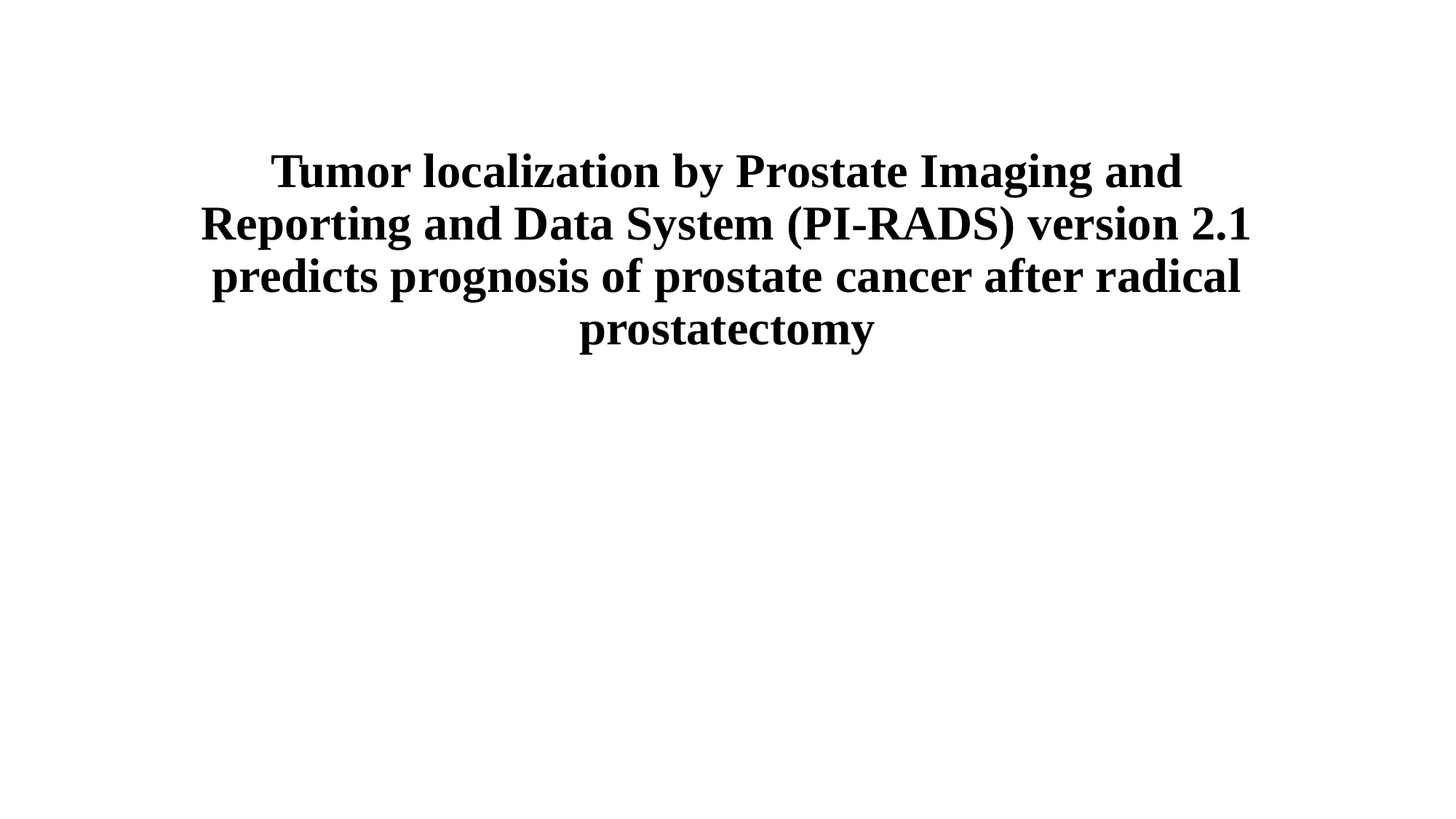

# Tumor localization by Prostate Imaging and Reporting and Data System (PI-RADS) version 2.1 predicts prognosis of prostate cancer after radical prostatectomy

## Slide 2
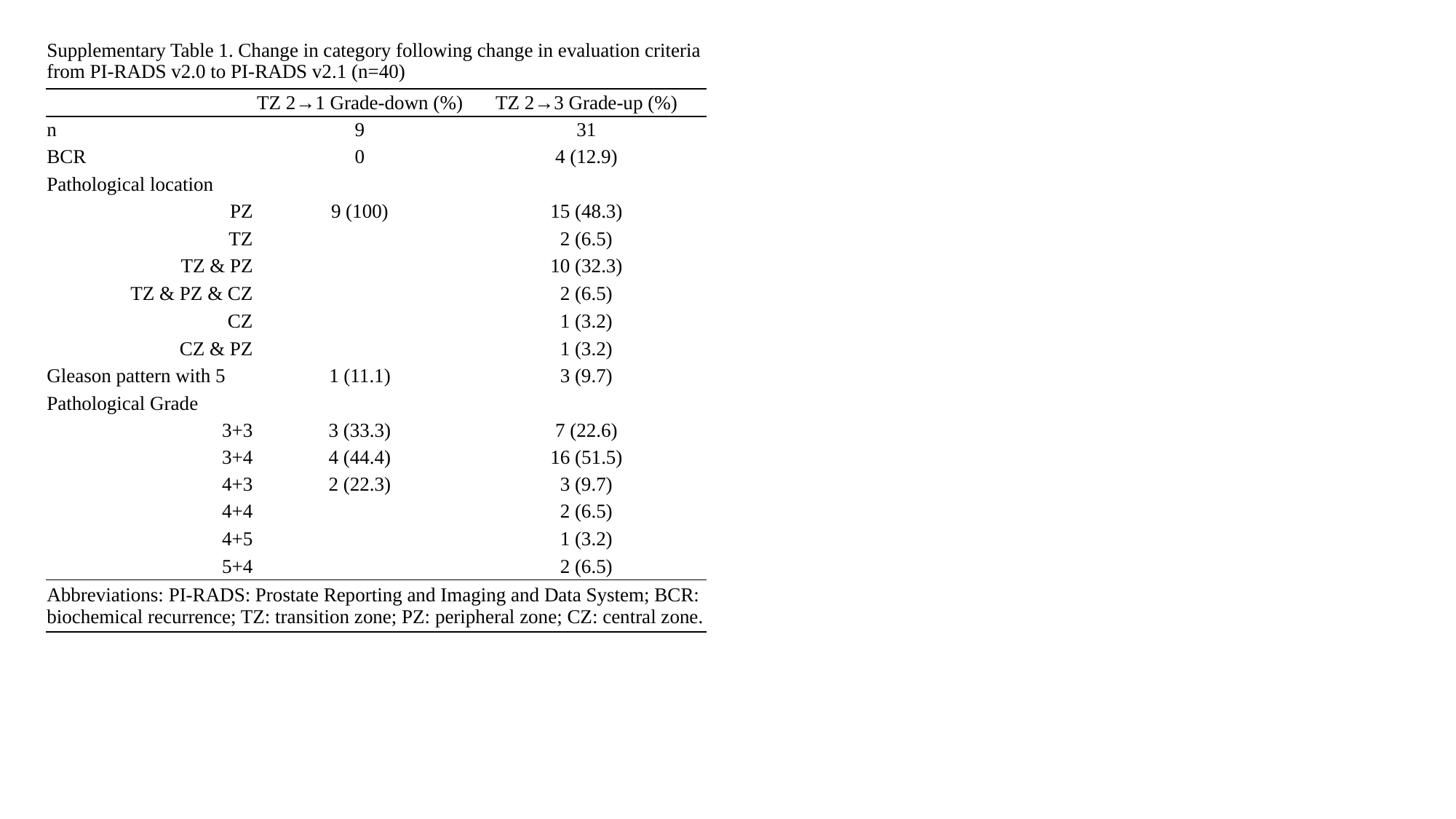

| Supplementary Table 1. Change in category following change in evaluation criteria from PI-RADS v2.0 to PI-RADS v2.1 (n=40) | | | |
| --- | --- | --- | --- |
| | TZ 2→1 Grade-down (%) | TZ 2→3 Grade-up (%) | |
| n | 9 | 31 | |
| BCR | 0 | 4 (12.9) | |
| Pathological location | | | |
| PZ | 9 (100) | 15 (48.3) | |
| TZ | | 2 (6.5) | |
| TZ & PZ | | 10 (32.3) | |
| TZ & PZ & CZ | | 2 (6.5) | |
| CZ | | 1 (3.2) | |
| CZ & PZ | | 1 (3.2) | |
| Gleason pattern with 5 | 1 (11.1) | 3 (9.7) | |
| Pathological Grade | | | |
| 3+3 | 3 (33.3) | 7 (22.6) | |
| 3+4 | 4 (44.4) | 16 (51.5) | |
| 4+3 | 2 (22.3) | 3 (9.7) | |
| 4+4 | | 2 (6.5) | |
| 4+5 | | 1 (3.2) | |
| 5+4 | | 2 (6.5) | |
| Abbreviations: PI-RADS: Prostate Reporting and Imaging and Data System; BCR: biochemical recurrence; TZ: transition zone; PZ: peripheral zone; CZ: central zone. | | | |
